# Administration of 4-(*α*-L-Rhamnosyloxy)-benzyl Isothiocyanate Delays Disease Phenotype in SOD1^G93A^ Rats: A Transgenic Model of Amyotrophic Lateral Sclerosis

**DOI:** 10.1155/2015/259417

**Published:** 2015-05-05

**Authors:** Maria Galuppo, Sabrina Giacoppo, Renato Iori, Gina Rosalinda De Nicola, Placido Bramanti, Emanuela Mazzon

**Affiliations:** ^1^IRCCS Centro Neurolesi “Bonino-Pulejo”, Via Provinciale Palermo, Contrada Casazza, 98124 Messina, Italy; ^2^Council for Agricultural Research and Economics-Research Centre for Industrial Crops (CRA-CIN), Via di Corticella 133, 40128 Bologna, Italy

## Abstract

4-(*α*-L-Rhamnosyloxy)-benzyl glucosinolate (glucomoringin, GMG) is a compound found in *Moringa oleifera* seeds. Myrosinase-catalyzed hydrolysis at neutral pH of GMG releases the biologically active compound 4-(*α*-L-rhamnosyloxy)-benzyl isothiocyanate (GMG-ITC). The present study was designed to test the potential therapeutic effectiveness of GMG-ITC to counteract the amyotrophic lateral sclerosis (ALS) using SOD1tg rats, which physiologically develops SOD1^G93A^ at about 16 weeks of life, and can be considered a genetic model of disease. Rats were treated once a day with GMG (10 mg/Kg) bioactivated with myrosinase (20 *µ*L/rat) via intraperitoneal (i.p.) injection for two weeks before disease onset and the treatment was prolonged for further two weeks before the sacrifice. Immune-inflammatory markers as well as apoptotic pathway were investigated to establish whether GMG-ITC could represent a new promising tool in clinical practice to prevent ALS. Achieved data display clear differences in molecular and biological profiles between treated and untreated SOD1tg rats leading to guessing that GMG-ITC can interfere with the pathophysiological mechanisms at the basis of ALS development. Therefore, GMG-ITC produced from myrosinase-catalyzed hydrolysis of pure GMG could be a candidate for further studies aimed to assess its possible use in clinical practice for the prevention or to slow down this disease.

## 1. Introduction

Amyotrophic lateral sclerosis (ALS) is a motor neuron disease first described by Jean-Martin Charcot in the 1800s. The disease gradually and fatally attacks both the first motor neurons in the cerebral cortex and the second motor neurons in the brainstem and spinal cord (the upper and lower motor neuron, resp.) responsible for controlling the voluntary muscles. It is a neurodegenerative pathology with a progressive and invariably fatal outcome. The disease is sometimes called Lou Gehrig's disease and, less frequently, Charcot disease [1]. Sadly, as a neuromuscular disease, it is related just to the motor system so that all the neurological functions are preserved and the patient is wholly aware of what is happening.

Mostly, ALS onset occurs in late adulthood [2] starting in limb, axial, bulbar, or respiratory muscles and causing spasticity and severe and rapidly progressive muscle weakness and respiratory insufficiency that lead to death within few years after initial diagnosis.

Sporadic (sALS) and familial (fALS) forms of the disease represent, respectively, about 90% and 10%, of all ALS cases [3].

To date the causes are unknown; nevertheless it is believed that ALS could have a multifactorial etiology, where environmental factors can greatly contribute to pathology triggering [4]. Both a defect in glutamate transporter and calcium binding protein failure have been identified as potential causes of the corticomotoneuronal system defects [5]. Moreover, genetic mutations on chromosome 21, which codes for the cytosolic antioxidant enzyme Cu2+/Zn2+ binding superoxide dismutase gene 1 (SOD1), have been identified first as a cause associated with 20% of all familial forms [5]. Other possible genetic causes have been related to defects in transactivation response (TAR) element, DNA-binding protein 43 (TDP-43), angiogenin, and an intronic hexanucleotide expansion in the gene encoding the chromosome 9 open reading frame 72 (*C9orf72*) [2].

Also potentiated to be involved in the genesis of the disease is the role of the innate immune system with cellular mechanisms mediated by CD8+ cytotoxic cells and CD4+ T-helper cells localized in spinal cord ventral horns, in the anterior and lateral corticospinal tracts and in the motor cortex [2].

The most commonly used drug to treat ALS is glutamate-antagonist riluzole that prolongs patient survival but has very limited benefits, since it does not show the expected efficacy that could lead to disease resolution. For this reason, new innovative and safer therapies are needed [6, 7], at least aimed at delaying the neurodegenerative processes of the ongoing disease.

In an effort to discover new active compounds as alternatives to the current therapies, basic science is focused on natural products and their derivatives for the treatment of neurodegenerative diseases, such as ALS [8, 9].

**Figure 1:**
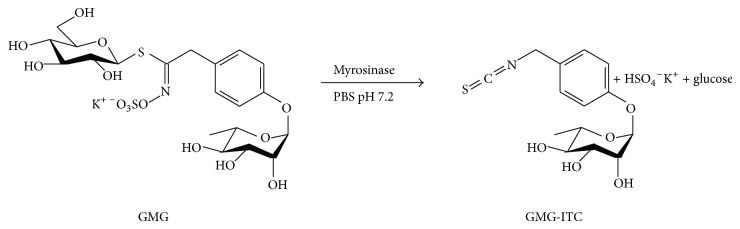
Chemical structures and production of GMG-ITC. Myr-catalyzed hydrolysis of 4-(*α*-L-rhamnosyloxy)-benzyl glucosinolate (glucomoringin, GMG) to produce 4-(*α*-L-rhamnosyloxy)-benzyl isothiocyanate (GMG-ITC).

Recently, our two research groups have produced evidence about the beneficial effects of isothiocyanates. These compounds are released by hydrolysis of precursor glucosinolates by myrosinase (Myr; *β*-thioglucoside glucohydrolase; E.C. 3.2.1.147). In particular, hydrolysis at neutral pH of 4-(*α*-L-rhamnosyloxy)-benzyl glucosinolate (commonly named glucomoringin, GMG) by Myr produces 4-(*α*-L-rhamnosyloxy)-benzyl isothiocyanate (GMG-ITC), a bioactive glycosylated isothiocyanate (Figure 1).

GMG was firstly purified by one of the authors in 2000 and using a synthetic sequence the structure of this glucosinolate extracted from* M. oleifera* seeds was unequivocally ascribed and fully characterized by ^1^H and ^13^C NMR and mass spectrometry [10, 11].

GMG is a secondary metabolite present in large amounts in* Moringa oleifera* seeds [12] and recently has been easily purified on a gram scale at the Bologna laboratory (CRA-CIN; previously named Istituto Sperimentale per le Colture Industriali) using an established method [13, 14].

*M. oleifera* is the most widely distributed plant of* Moringa* genus and grows in many tropical and equatorial regions. The species is native to the dry tropical areas in Northwestern India at the Southwestern foot of the Himalaya.* M. oleifera* plant is used for human and animal nutrition as well as for a multitude of medicinal purpose [15]. GMG-ITC has shown a wide multiplicity of pharmacological properties ranging from neuroprotective capability to counteract secondary damage following spinal cord injury [16] up to the power to inhibit the cascade of events that is at the basis of experimental autoimmune encephalomyelitis [17].

For this reason, the aim of the present work was to test the potential effectiveness of GMG-ITC to interfere with the mechanisms underlying ALS development and prevent or slow down the disease in transgenic SOD1^G93A^ (SOD1tg) rats, an experimental genetic model of developing disease.

## 2. Materials and Methods

### 2.1. Animals

Male Sprague-Dawley rats overexpressing the mutated human gene SOD1^G93A^, which represents a transgenic model of ALS, were purchased from Taconic Biosciences, Inc. (Hudson, NY, USA) and used for the experiment.

### 2.2. ALS Model of Disease

According to genetic and phenotypic description provided by Taconic industry (http://www.taconic.com/2148) spinal cord of SOD1G93A hemizygous rats expresses about 8-fold more endogenous SOD1, which became ~16-fold by the end stages of disease. SOD1 rats have an onset of motor neuron disease after approximately 115 days.

### 2.3. GMG and Myrosinase Purification

**Figure 2:**
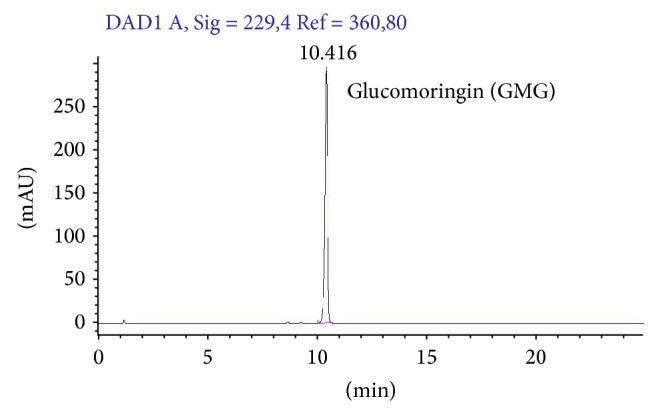
GMG chromatogram. HPLC chromatogram of purified 4-(*α*-L-rhamnosyloxy)-benzyl glucosinolate (glucomoringin, GMG).

GMG was isolated from* M. oleifera* L. (fam. Moringaceae) seeds (cake powder PKM2 provided by Indena India Pvt. Ltd; Bangalore, India) in two sequential steps, by anion exchange and size exclusion chromatography, according to previously reported methods [13, 14]. The cake powder PKM2 was treated with boiling 70% ethanol in order to quickly deactivate the endogenous enzyme myrosinase. GMG was extracted using an Ultraturrax homogenizer at a medium speed for 15 min and the resulting homogenate was centrifuged at 17,700 ×g for 30 min. The isolation of GMG from the extract was carried out by one-step anion exchange chromatography. The extract was loaded on a DEAE-Sephadex A-25 (GE Healthcare, Milan, Italy) anion-exchange column (150 × 26 mm) conditioned with 25 mM acetate buffer (pH 5.6). After washing with 1 L of distilled water, GMG was eluted with 500 mL of aqueous K_2_SO_4_ 0.2 M. The eluate was concentrated to dryness using a rotary evaporator at 60–70°C under vacuum. Three subsequent extractions were carried out with 70–100 mL of boiling methanol. Then, the alcoholic extract was filtered and concentrated to 15–20% of the initial volume. The solution was warmed and slowly added dropwise to 200 mL of ethanol that was previously cooled to −20°C. This led to the precipitation of a white powder. After centrifugation, the solid GMG (as potassium salt) was dried and sealed under vacuum to prevent moisture uptake by the highly hygroscopic compound. The purity of the GMG was further improved by gel-filtration performed using an XK 26/100 column packed with Sephadex G10 (GE Healthcare, Milan, Italy) connected to a fast protein liquid chromatograph system (AKTA FPLC System, GE Healthcare, Milan, Italy). The mobile phase was water at a flow rate of 2.0 mL min^−1^, and the eluate absorbance was monitored at 254 nm. After the void volume was discarded, 5 mL fractions were collected and analysed by HPLC and those containing pure GMG were pooled and freeze-dried. The purity was assayed by HPLC analysis of the desulfo-derivative according to the ISO 9167-1 method [18] yielding about 99% based on peak area value (Figure 2), and more than 95% on weight basis, due to its high hygroscopic properties, was determined by a calibration curve of standard desulfo-GMG available in our laboratory. The enzyme Myr was isolated from seeds of* Sinapis alba* L. according to a reported method with some modifications [19].

Briefly, the enzyme was extracted from white mustard seeds with water and purified by affinity chromatography on Con A-Sepharose. Then, the active fractions coming from affinity chromatography were pooled and dialyzed against 50 mM phosphate buffer pH 6.5 containing 0.15 M NaCl. The dialyzed Myr solution was concentrated and loaded into a prepacked Superdex 200 HiLoad 26/60 gel filtration column (GE Healthcare) equilibrated with 50 mM phosphate buffer pH 6.5 containing 0.15 M NaCl connected with a fast protein liquid chromatograph system (AKTA FPLC System, GE Healthcare, Milan, Italy). The active fractions were pooled and concentrated by Millipore Amicon Stirred Cell Model 8400 using a UF membrane 30 KDa MWCO (Millipore). The stock solution used in the present study had a specific activity of 60 units/mg of soluble protein. The enzymatic activity was 32 U/mL and the solution was stored at 4°C in sterile saline solution at neutral pH until use. One Myr unit was defined as the amount of enzyme able to hydrolyze 1 *μ*mol/min of sinigrin at pH 6.5 and 37°C.

### 2.4. Myr-Catalyzed Hydrolysis of GMG and Animal Treatment

**Figure 3:**
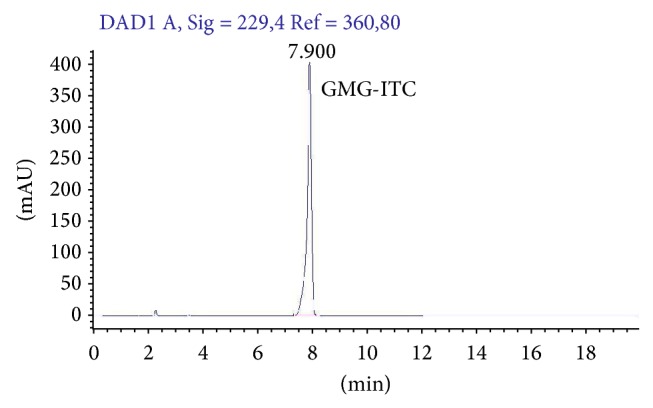
GMG-ITC chromatogram. HPLC chromatogram of 4-(*α*-L-rhamnosyloxy)-benzyl isothiocyanate (GMG-ITC) produced by myrosinase catalyzed hydrolysis of purified 4-(*α*-L-rhamnosyloxy)-benzyl glucosinolate (glucomoringin, GMG).

GMG was dissolved in PBS solution pH 7.2 at room temperature and immediately before rat intraperitoneal (i.p.) treatment, the action of Myr (10 mg/Kg GMG + 20 *μ*L enzyme/rat; 1.0 mL) on GMG was allowed to take place for 15 min at 37°C (see Figure 1 for in vitro reaction of GMG-ITC production). The total conversion of pure GMG into GMG-ITC, before animal administration, was confirmed by HPLC analysis (Figure 3) using an Agilent Model 1100 HPLC system with an Inertsil ODS3 column (250 mm × 3 mm, 5 *μ*m). Chromatography was performed with 1 mL/min flow rate at 30°C by eluting with a linear gradient of water (A) and acetonitrile (B) from 30% B to 80% in 20 min, monitoring the absorbance at 229 nm.

### 2.5. Experimental Groups

Rats were randomly allocated into the following groups:

- Untreated SOD1tg group (*n* = 10): rats not pharmacologically treated.
- GMG-ITC-treated SOD1tg group (*n* = 10): rats were prophylactically treated once a day with GMG (10 mg/Kg) bioactivated with Myr (20 *μ*L/rat) as described above, via i.p. injection starting from two weeks before the disease onset (about 100 days of life) and protracted for other two weeks before the sacrifice (about 130 days of life).

Since the Taconic industry disease onset occurs around 115 days of life (about 16 weeks), our experiments were planned to treat SOD1tg rats starting from 14 weeks. Consequently, the sacrifice was established when in the GMG-ITC SOD1tg group are appeared the first signs of disease (about 18 weeks of life).

At the end of the experiment, blood was collected by cardiac puncture and animals were euthanized. Brain tissue and spleen were sampled and processed in order to evaluate disease parameters.

### 2.6. Behavioral Tests

Noninvasive behavioral evaluations were made without causing excessive animal stress in order to provide data about muscular degeneration/locomotor activity loss.

Hanging Wire Test (HWT) was performed to evaluate motor performance. The test consists in the capability of the animal into staying hanged to a wire for a time period of 90 sec, using the paw strength. HWT was performed two times a week starting from four weeks before the disease onset and two weeks later and for a total number of fifteen tests.

Also Open Field Test (OFT) for motor function was performed to test behavior and general motor function. GMG-ITC treated as well as untreated SOD1tg rats were monitored for a time period of 180 sec to assess the spontaneous activity in an open field, consisting of a white Plexiglas box (100 cm × 100 cm) with the floor divided into 16 squares. Four squares were defined as the center and 12 squares along the walls as the periphery. Each animal was gently placed in the center of the box and activity was scored as a line crossing when a mouse removed all four paws from one square and entered another. Immediately after each test, the apparatus was thoroughly cleaned with cotton pad wetted with 70% ethanol. The test was performed once a week starting from two weeks before the disease onset and two weeks later, with the last measure performed the day before the sacrifice and for a total number of six tests.

### 2.7. IHC Localization

Brain tissues were fixed in 10% (w/v) PBS-buffered formaldehyde, and 6 *μ*m sections were prepared from paraffin-embedded tissues. After deparaffinization, endogenous peroxidase was quenched with 0.3% (v/v) hydrogen peroxide in 60% (v/v) methanol for 30 min. Nonspecific adsorption was minimized by incubating sections in 2% (v/v) normal goat serum in PBS for 20 min. Endogenous biotin or avidin binding sites were blocked by sequential incubation for 15 min with biotin and avidin, respectively.

Sections were incubated overnight with the following primary antibodies:

- Anti-TLR4 monoclonal antibody (1 : 100 in PBS v/v; Abcam).
- Anti-MMP9 polyclonal antibody (1 : 100 in PBS v/v; Abcam).
- Anti-NOS2 polyclonal antibody (1 : 100 in PBS v/v; Santa Cruz Biotechnology, Inc).
- Anti-PARP-1 polyclonal antibody (1 : 100 in PBS v/v; Santa Cruz Biotechnology, Inc).
- Anti-CD8*α* polyclonal antibody (1 : 100 in PBS v/v; Santa Cruz Biotechnology, Inc).
- Anti-Nrf2 polyclonal antibody (1 : 100 in PBS v/v; Santa Cruz Biotechnology, Inc).

Sections were washed with PBS and incubated with secondary antibody. Specific labeling was detected with a biotin-conjugated goat anti-rabbit IgG and avidin-biotin peroxidase complex. The counterstain was developed with diaminobenzidine (brown color) and ematossilin (blue background).

To verify the binding specificity, some sections were also incubated with only the primary antibody or with only the secondary antibody. In these situations, absence of positive staining was found in the sections, indicating that the immunoreaction was positive in all the experiments carried out.

All sections were observed using light microscopy (Leica ICC50 HD).

Leica Application Suite V4.2.0 software was used as image computer program to acquire IHC pictures.

### 2.8. Western Blot Analysis

All western blot procedures aimed to assess the expression of mediators of ALS development were performed according to previously published protocols [17] and here modified for brain tissue and spleen.

Briefly, all the extraction procedures were performed on ice using ice-cold reagents. Brain tissues or spleen were suspended in extraction buffer containing 0.32 M sucrose, 10 mM Tris-HCl, pH 7.4, 1 mM EGTA, 2 mM EDTA, 5 mM NaN3, 10 mM 2-mercaptoethanol, 50 mM NaF, and protease inhibitor tablets (Roche, Milan, Italy) and then homogenized at the highest setting for 2 min. The homogenates were chilled on ice for 15 min and then centrifuged at 1000 g for 10 min at 4°C, and the supernatant (cytosol + membrane extract) was collected to evaluate content of citoplasmatic proteins.

The pellets were suspended in the supplied complete lysis buffer containing 1% Triton X-100, 150 mM NaCl, 10 mM Tris-HCl, pH 7.4, 1 mM EGTA, and 1 mM EDTA protease inhibitors tablets (Roche), and then they were centrifuged for 30 min at 15,000 g at 4°C, and the supernatant (nuclear extract) was collected to evaluate the content of nuclear proteins.

Supernatants were stored at −80°C until use. Protein concentration in homogenate was estimated by Bio-Rad Protein Assay (Bio-Rad, Segrate, Italy) using BSA as standard, and 20 *μ*g of cytosol and nuclear extract from each sample were analyzed.

Proteins were separated on sodium dodecyl sulfate-polyacrylamide minigels and transferred onto nitrocellulose membranes (Protran nitrocellulose transfer membrane; Whatman Schleicher and Schuell, Dassel, Germany), blocked with PBS containing 5% nonfat dried milk (PM) for 45 min at room temperature, and subsequently probed at 4°C overnight with the follow primary antibodies: TNF-alpha (1 : 100 in PM 0.1% Tween 20 (PMT) v/v; Cell Signaling Technologies), FoxP3 (1 : 500 in PMT v/v; eBioscience), and cleaved-caspase3 (1 : 1000 in PMT v/v; Cell Signaling Technologies).

HRP-conjugated goat anti-mouse IgG or HRP-conjugated goat anti-rabbit IgG were incubated as secondary antibodies (1 : 2000 in PMT v/v; Santa Cruz Biotechnology, Inc.) for 1 h at room temperature. To ascertain that blots were loaded with equal amounts of proteic lysates, they were also incubated with GAPDH-HRP conjugated primary antibody (1 : 1000 in PMT v/v; Cell Signaling Technology).

Relative protein bands expression was visualized using an enhanced chemiluminescence system (Luminata Forte, Western HRP substrate, Millipore) and proteic bands were acquired and quantified with ChemiDoc MP System (Bio-Rad, Segrate, Italy) and a computer program (ImageJ software), respectively.

Blots are representative of three separate and reproducible experiments.

Statistical analysis was run on three repeated blots performed on separate experiments.

### 2.9. Blood Sampling

At the sacrifice, blood samples were collected via cardiac puncture in Serum Separator Tubes (Vacutainer SSTTM II Advance, BD Diagnostic, Milan, Italy) and centrifuged following at least 30 min from the collection at 2000 g speed for 10 min. The achieved serum was collected, aliquoted, and stored at −80°C to be used in next investigations.

### 2.10. Prostaglandin E2 (PGE2) Assay

ELISA kit for PGE2 parameter assay (R&D system Europe, Ltd., Abingdon, UK) was purchased to detect PGE2 levels in serum samples. The kit was used according to the manufacturer's instruction and achieved O.D. were tabulated and analyzed using a software of elaboration data.

### 2.11. Chemical Serum Parameters

VITROS MicroSlide Tests (VITROS 350 by Ortho-Clinical Diagnostics, Johnson & Johnson company, Milan, Italy) were used to assess creatine kinase (CK), sodium (Na+), and potassium (K+) serum levels. Data are shown as mean of achieved values for each experimental group.

### 2.12. Golgi Stain

FD Neurotech kit (FD NeuroTechnologies, Ellicott City, Md, USA) was used for Golgi impregnation of tissue, according to manufacturing instruction (http://fdneurotech.com/docs/1333571253.web_pk401-401a-04042012.pdf).

Briefly, brain samples were placed directly into solution (A + B) containing mercuric chloride, potassium dichromate, and potassium chromate, without rinsing, and remained there for 2 weeks in the dark at room temperature. Forty-eight hours after placing in solution C (4°C), brains were frozen on dry ice and stored at −70°C until sectioning. Cryostat sections (100 *μ*m) were cut at −25°C and mounted onto gelatinized slides. Slides were allowed to dry in the dark, and the rest of the staining process was done as previously described [20]. Cresyl violet was used as background color to counterstain.

### 2.13. Statistical Analysis

All data were elaborated using GraphPad Prism version 6.0 (GraphPad Software, La Jolla, CA). The results were statistically analyzed by performing Student's *t*-test. A *p* value of < 0.05 was considered to be statistically significant. Results are expressed as mean ± S.E.M. of *n* experiments.

For behavioral assessment, Sidak-Bonferroni method was applied using multiple *t*-tests to assess statistical differences.

## 3. Results and Discussion

The glycosylated isothiocyanate GMG-ITC, resulting from Myr-hydrolysis (>99%) of GMG at neutral pH value (Figure 1), has been shown to exhibit a broad range of biological activity including effective antitumor activity [21]. While previous research has reported that GMG-ITC is more stable than other ITCs such as sulforaphane from broccoli [22], we administrated GMG-ITC to rats immediately after its production to avoid its decomposition in solution.

SOD1tg rats represent a genetic model of ALS. Our purpose was to verify whether GMG-ITC could have some effects on ALS disease and, if so, to investigate the mechanism which was modulated by pharmacological treatment of animals starting two weeks before the disease onset. In particular, we aimed to assess whether GMG-ITC treatment could shift forward the time of disease onset, characterized by hind limb abnormal gait associated with degeneration of muscle integrity and function. It was evident that the appearance of muscle spasticity and abdominal contortion were initially visible in untreated rats SOD1tg (16 weeks of life in untreated SOD1tg rats) and at a later time, about two weeks of delay (18 weeks of life), in rats treated with GMG-ITC.

**Figure 4:**
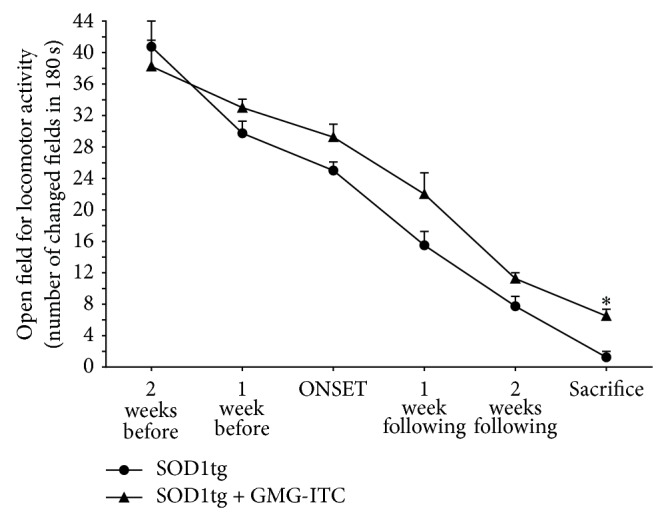
GMG-ITC treatment delays locomotor activity loss. OFT was performed to evaluate SOD1tg rats motility in an open field, as a result of the administration or no administration of GMG-ITC treatment. Following seven measures, rats that received GMG-ITC showed higher locomotor activity for the whole observational period with a significant difference at the day before the sacrifice. ^∗^ *p* = 0.003.

Behavioral tests were a helpful tool in monitoring disease progression and GMG-ITC treatment effects. In particular, performing OFT, we established a locomotor activity of GMG-ITC treated SOD1tg rats in the arena higher than untreated SOD1tg rats (Figure 4). It is noteworthy to mention that even if the motility of GMG-ITC SOD1tg rats appears to decrease in the time, the last measure occurring the day before the sacrifice shows a significant difference between the two groups, leading to believe that, however, GMG-ITC treatment has the capability to delay deficits by ALS.

**Figure 5:**
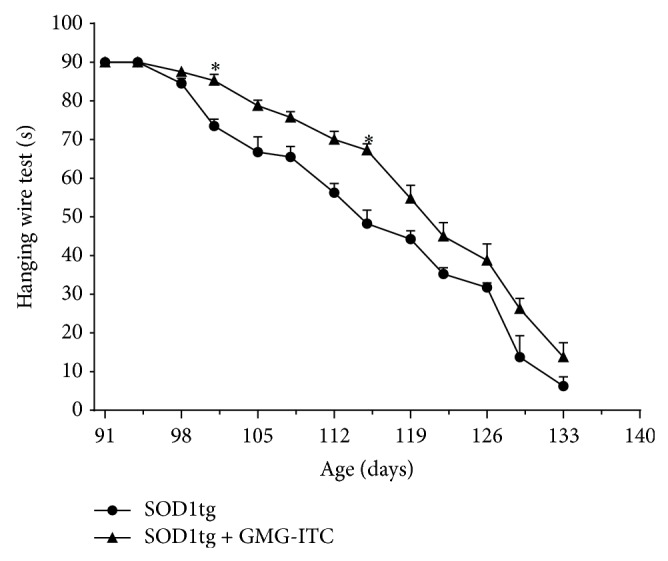
GMG-ITC treatment delaying ALS progression maintains muscle efficiency. HWT displays an initial overlapping of performance between the two experimental groups. Nevertheless, starting from forth test significant differences occur, showing better muscle strength and attitude in GMG-ITC-treated SOD1tg rats in all tests made during the experimental period. ^∗^ *p* = 0.002.

Also, WHT, often used in place of rotarod test to assess the natural course of neuromuscular disease, allowed to establish a better performance of SOD1tg rats treated with GMG-ITC given by a stronger grip capability and a better muscle strength, although decreasing over time (Figure 5).

To understand the underlying molecular and cellular mechanisms we looked at the activation of the innate and acquired immune system, since it is inextricably related to many neurodegenerative and neuromuscular diseases [2, 23].

**Figure 6:**
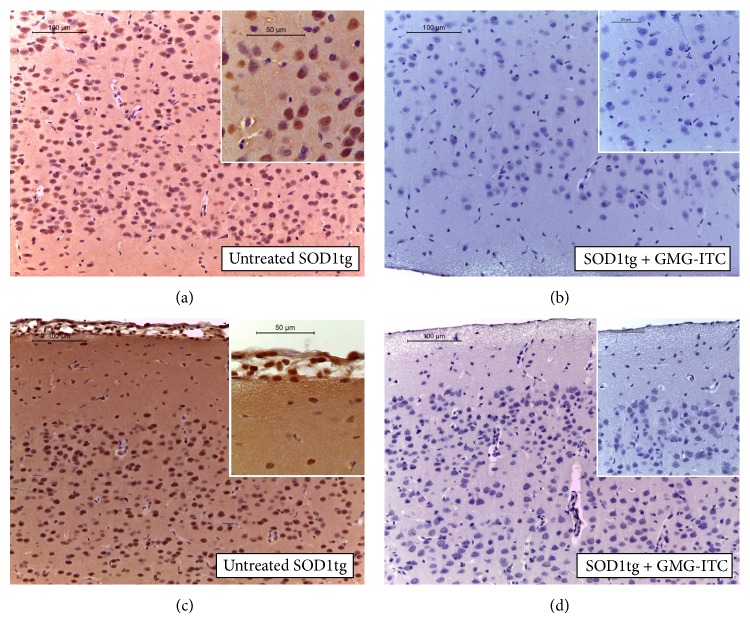
GMG-ITC treatment modulates innate and acquired immune response. In brain sections, TLR-4 detection reveals immunopositivity in untreated SOD1tg rats (a), while SOD1tg rats treated with GMG-ITC show negative staining for TLR4 (b). In untreated SOD1tg rats the immunopositivity of brain sections to CD8 antibody identified wide areas with infiltrating cells (c). GMG-ITC treatment reveals the capability to counteract the release of cytotoxic T cells at level of brain sections (d).

As expected, we observed that both TLR4 and CD8*α* detections were apparent in untreated SOD1tg rats (Figures 6(a) and 6(c), resp.; see densitometric analysis Figure 12). Conversely, the effects of GMG-ITC produced an immunomodulatory action reducing immune-competent cell solicitation (Figures 6(b) and 6(d), resp.; see densitometric analysis Figure 12).

**Figure 7:**
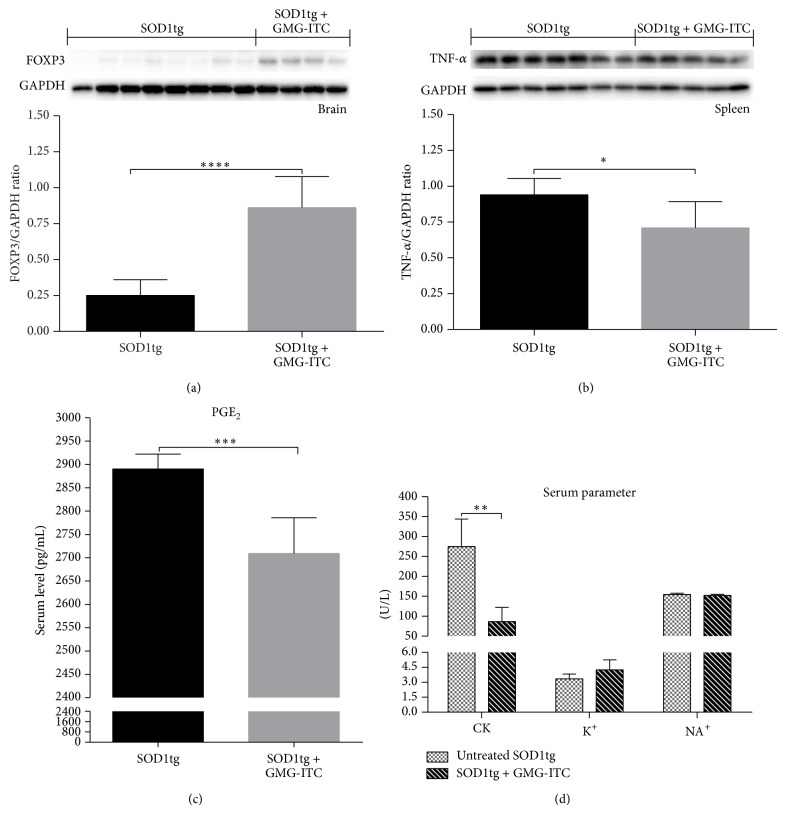
Western blot analysis of FoxP3 and TNF-*α* expression and serum parameters. Brain protein extracts reveal significant differences. GMG-ITC treated SOD1tg rats present levels of FoxP3 higher than untreated animals (a) and TNF-alpha expression in spleen homogenates shows significant higher level of this marker in untreated than GMG-ITC treated SOD1tg rats (b). ^∗∗∗∗^ *p* < 0.0001; ^∗^ *p* = 0.0223. PGE2 serum levels are significantly higher in untreated SOD1tg rats when compared with serum levels of animals treated with GMG-ITC (c). ^∗∗∗^ *p* = 0.0001. About serum parameters, no electrolyte imbalance was measured in both groups looking at NA+/K+ levels, when creatine kinase (CK) enzyme was assayed, while a significant and interesting change was found comparing the CK levels of untreated SOD1tg rats higher than GMG-ITC treated animals (d). ^∗∗^ *p* = 0.008.

These data have been further validated in GMG-ITC treated SOD1tg rat by a high and significative FoxP3 detection, as an indirect marker of Tregulatory (Treg, also known as CD4^+^/CD25^high^/FoxP3^+^) cell presence (Figure 7(a)). Treg cell recruitment plays a key defensive role in suppression of Th1 effector cells [24], which are the main T cell subtype mediating disease pathogenesis. Interestingly, it is possible that GMG-ITC stimulates Th0 cell to develop into a Treg phenotype. Moreover, looking at proinflammatory cytokine profile classically activated by microglia during ALS development [25], levels of TNF-*α* result significantly decreased following GMG-ITC treatment (Figure 7(b)). Taken together, all these parameters suggest that there is an upstream regulation of immune-inflammatory mediators as confirmed by increased PGE2 serum levels in the untreated SOD1tg rats [26] and by the modulation of this marker following GMG-ITC administration (Figure 7(c)). Furthermore, the influence that the prophylactic administration of GMG-ITC has on ALS development is evident assessing CK activity, an enzyme measured to monitor muscular deficit and atrophy and characteristically altered in ALS patients [27]. In fact, while no variation was detected in electrolytic balance between treated and untreated SOD1tg rats, a marked difference in serum CK was found in GMG-ITC treated SOD1tg rats that show lower levels. This data demonstrated the capability of GMG-ITC to interfere with motor neuron degeneration blocking radical species production, which is at the basis of many neurodegenerative diseases, including ALS [26] (Figure 7(d)).

**Figure 8:**
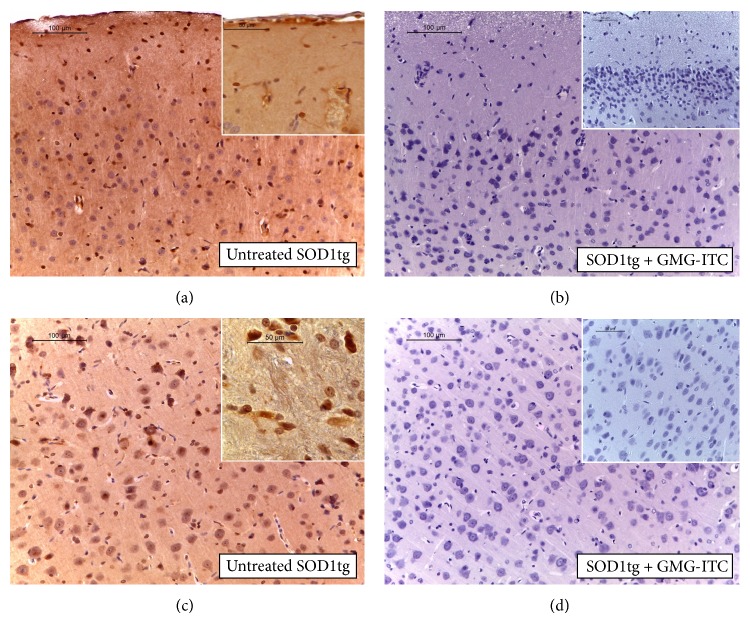
iNOS formation and MMP-9 expression. Immunohistochemical (IHC) detection of iNOS tissue expression reveals that untreated SOD1tg rats have a positive staining for this marker (a), while there is an absence of positivity in brain sections sampled from GMG-ITC treated SOD1tg rats (b). By IHC stain, brain sections sampled from untreated SOD1tg rats exhibit positive staining for MMP-9 (c), whereas brain sections obtained from GMG-ITC treated SOD1tg rats do not stain for MMP-9 (d).

Our data suggest that the high component of radicalic species is the cause of neuromuscular degeneration leading to ALS development. In this regard, it was interesting to investigate both the capability of GMG-ITC to preserve brain tissue by oxidative stress and the state of the zinc-dependent endopeptidase MMP9. Overall, data in literature show that this enzyme is not specific for ALS; nevertheless it is associated with ALS as marker of pathogenesis exerting direct neurotoxic effects or causing death by matrix proteins degradation [28]. Convincing data about iNOS expression (Figure 8(a) versus Figure 8(b); see densitometric analysis Figure 12) as well as MMP-9 detection (Figure 8(c) versus Figure 8(d); see densitometric analysis Figure 12) have been produced showing tissue preservation by ALS disease in rats treated with GMG-ITC.

**Figure 9:**
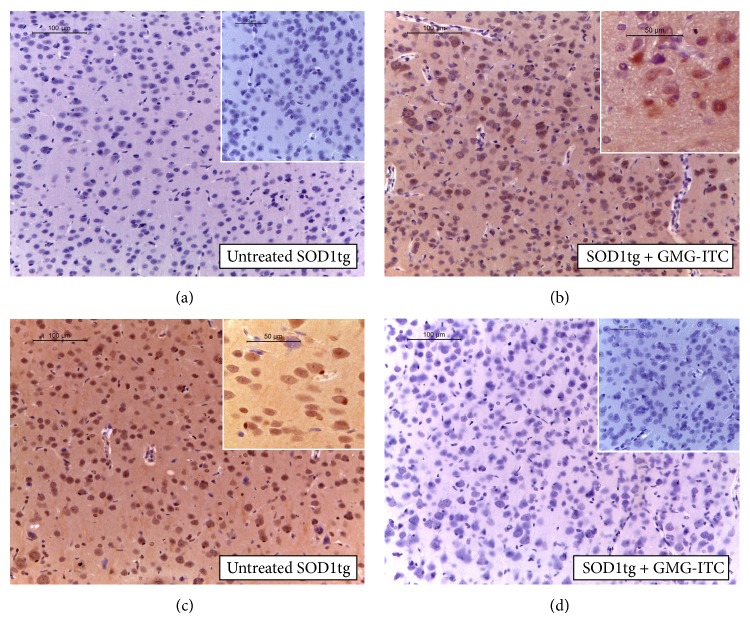
GMG-ITC treatment promotes Nrf-2 activity and reduces PARP-1 activity. Nrf2 IHC localization shows a negative expression in brain sections sampled from untreated SOD1tg rats (a), while GMG-ITC administration stimulates Nrf2 nuclear activity (b) preserving tissue damage by prooxidative gene expression. PARP-1 immunodetection shows a positive staining in untreated SOD1tg rats (c) and an IHC negative localization in GMG-ITC treated SOD1tg rats (d).

The mechanism by which GMG-ITC inhibits prooxidative genes nuclear expression seems to be controlled by a nuclear factor (erythroid-derived 2)-like 2(Nrf-2)-mediated action (Figure 9(a) versus Figure 9(b); see densitometric analysis Figure 12). Moreover, we investigated the apoptotic pathway through different markers to evaluate how GMG-ITC is able to preserve cells by dysfunction and death processes. In particular, Poly (ADP-ribose) polymerase 1 (PARP-1), which is responsible for DNA breakdown in apoptosis processes and correlated to ALS progression [29], was reduced in the GMG-ITC treated rats, establishing the capability of GMG-ITC to prevent tissue damage (Figure 9(c) versus Figure 9(d); see densitometric analysis Figure 12).

**Figure 10:**
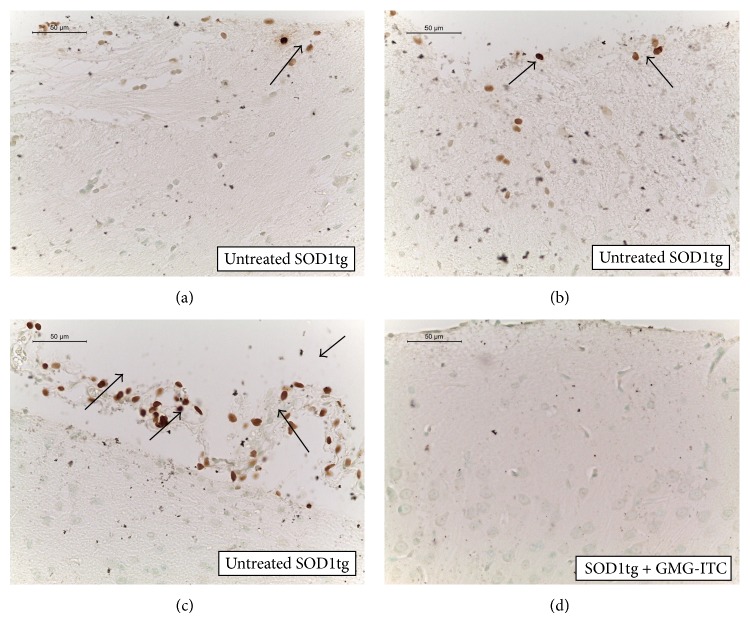
TUNEL assay for apoptosis detection. In untreated SOD1tg rats, black-brown apobodies are shown as an index of DNA breakdown (a, b, and c; see arrows). In brain sections sampled from GMG-ITC treated SOD1tg rats no apoptotic cells or fragments were present (d).

Also, protective effects of GMG-ITC in counteracting apoptosis are evaluable through the analysis of data regarding these markers and confirmed by the absence of apobodies in SOD1tg rats pharmacologically treated (Figures 10(a), 10(b), and 10(c) versus Figure 10(d)).

**Figure 11:**
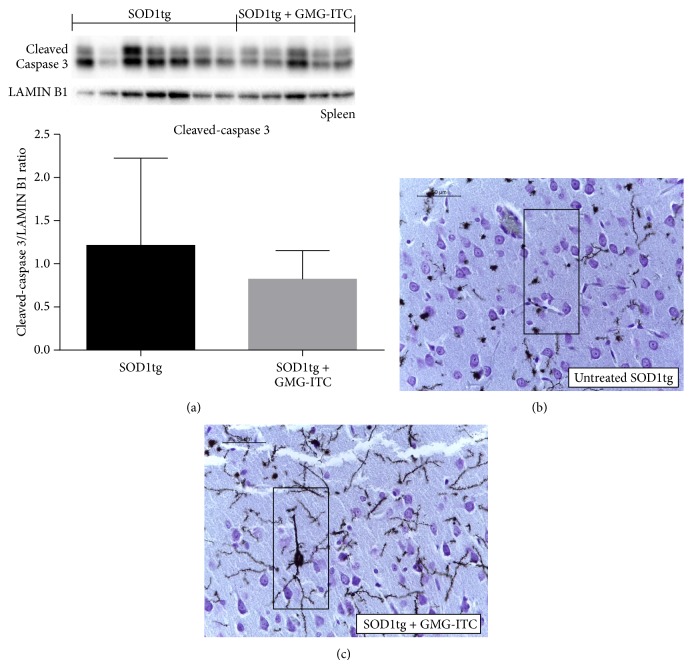
Cleaved-caspase3 activation and dendritic spine detection. Western blot analysis of spleen homogenates revealed that, although not significantly, cleaved-caspase3 levels are higher in untreated SOD1tg rats than in animals that received the GMG-ITC treatment (a). Moreover a complete loss of nerve processes was detected in untreated SOD1tg rats (b) while treatment with GMG-ITC protects SOD1tg rats neurons that appear morphologically intact and with long dendrites establishing normal synapses (c).

Supporting above cited data and adding further evidence about therapeutic effects of GMG-ITC, we found a modulated cleaved-caspase 3 activity in GMG-ITC-treated SOD1tg rats (Figure 11(a)), a mediator reported to influence PARP-1 expression [29]. Furthermore, also a preserved neuronal cell integrity was assessed in SOD1tg rats treated with GMG-ITC, resulting in normal and unaffected synaptic spine-spine communication at level of dendritic trees (Figure 11(b) versus Figure 11(c)). To compare dendritic loss, see areas in the rectangles.

**Figure 12:**
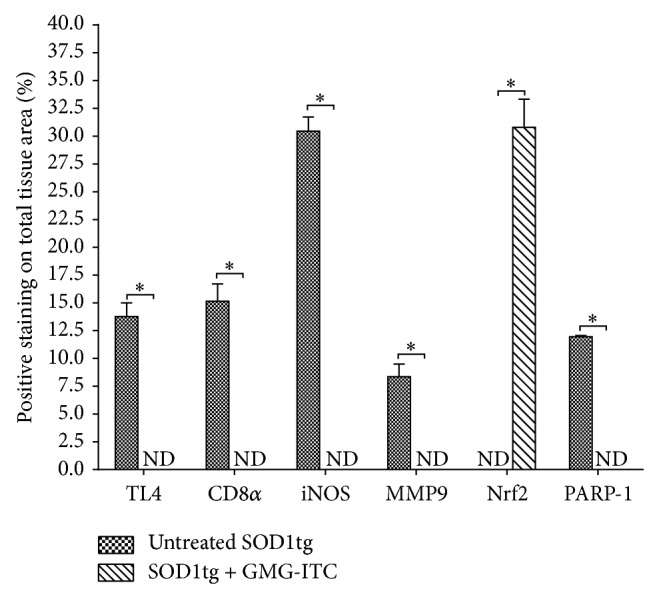
Densitometric analysis. Comparative expression between GMG-ITC treated/untreated SOD1tg rat for all evaluated immunohistochemical markers. A *p* value < 0.05 was considered significant. ND = not detectable.

Finally, providing a quantification of the above displayed immunopositivity, immunohistochemical images regarding TLR-4, CD8*α*, iNOS, MMP-9, Nrf-2, and PARP-1 were analyzed and the intensity was represented as % of positive staining (brown) on total tissue area (Figure 12).

## 4. Conclusions

Previous studies performed by our research group about GMG-ITC activity have revealed a wide range of effects and applications of the compound in many experimental pathological situations [16, 17, 30]. The present study adds a new promising use of the* Moringa oleifera* isothiocyanate in the treatment of a so severe pathology as is ALS. SOD1tg rats, which represent a transgenic model of this disease, showed a modified phenotype following GMG-ITC administration, displaying a delay in appearance of disease onset of about two weeks, and variations in serum parameters as well as in molecular and histochemical marker assessment. Overall, results support that GMG-ITC treatment possesses the capability to interfere with the mechanisms underlying ALS development. The bioactive phytochemical GMG-ITC, freshly produced by Myr-catalyzed hydrolysis of pure GMG, could be a candidate for further studies aimed to assess its possible use in clinical practice for the prevention or attenuated progression of ALS as well as other neuromuscular pathologies.
